# Laser therapy for genital lichen sclerosus: A systematic review of the current evidence base

**DOI:** 10.1002/ski2.52

**Published:** 2021-06-15

**Authors:** F. Tasker, L. Kirby, D. J. C. Grindlay, F. Lewis, R. C. Simpson

**Affiliations:** ^1^ King’s College London St John’s Institute of Dermatology London UK; ^2^ Centre of Evidence Based Dermatology University of Nottingham Nottingham UK

## Abstract

**Background:**

Lichen sclerosus (LS) is a chronic, inflammatory dermatosis. Initial treatment with superpotent topical corticosteroids is the accepted and evidence‐based first‐line therapy. For those who do not respond after exclusion of other potentiating factors, the best second‐line therapy is unclear. Laser therapy is an emerging treatment for genital LS and despite uncertain efficacy its use is gaining popularity in the private sector.

**Objectives:**

We aimed to review the effectiveness of laser therapy for genital LS in men, women and children.

**Methods:**

We conducted a systematic review of all primary studies reporting the use of laser in genital LS. Ovid MEDLINE, PubMed, Ovid Embase, Cochrane CENTRAL, Web of Science, CINAHL and PsycINFO were searched from inception to February 2021. The quality of the studies was assessed using the revised Cochrane risk‐of‐bias tool for randomized trials, ROBINS‐I tool for non‐randomized trials and Joanna Briggs Institute checklist for case studies.

**Results:**

A total of 24 studies, involving 616 adults, met inclusion criteria. These were six randomized controlled trials (RCTs), one non‐randomized trial, nine single arm trials and eight case series. Where assessed, most studies suggest that laser therapy in patients with LS may improve symptoms, clinical signs, quality of life and sexual function. However, results were highly heterogeneous and methodological quality was very low, therefore meta‐analysis was not possible.

**Conclusions:**

There is poor evidence to support the use of laser therapy for genital LS at present. Effectiveness of laser needs to be robustly investigated in well‐conducted RCTs.

## Graphical abstract

A super‐potent topical steroid is the evidence‐based first line therapy for genital lichen sclerosus. For those who do not respond to treatment, after exclusion of other potentiating factors, the best second‐line therapy is unclear. Laser therapy is an emerging treatment for genital lichen sclerosus and despite uncertain efficacy, its use is gaining popularity in the private sector. Our systematic review demonstrates that there is not enough evidence to recommend laser for the treatment of genital lichen sclerosus.

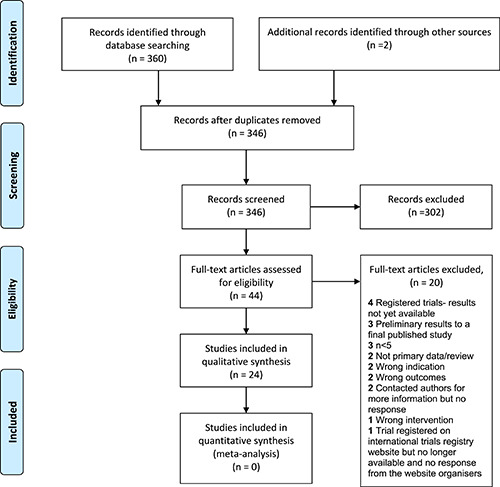

1

**What's already known about this topic?**

- A super‐potent topical steroid is the evidence‐based first line therapy for genital lichen sclerosus (LS).
- For those who do not respond to treatment, after exclusion of other potentiating factors, the best second‐line therapy is unclear.
- Laser therapy is an emerging treatment for genital LS and despite uncertain efficacy, its use is gaining popularity in the private sector.

**What does this study add?**

- There is not enough evidence to recommend laser for the treatment of genital LS.
- There is a lack of long‐term data on the procedure.

## 2 INTRODUCTION

Lichen sclerosus (LS) is a chronic, inflammatory dermatosis, with predilection for genital skin. The incidence of LS is 1.6% in women by age 80.^1^ Symptoms include intense itch, soreness and dyspareunia. Anatomical changes with resorption of the labia minora and fusion of the clitoral hood can occur. Scarring from dermal inflammation causes anatomical alteration. In males, LS typically occurs on the glans penis and foreskin, leading to phimosis, difficulty with micturition and dyspareunia.^2^ LS also affects children although this is less common. LS has considerable impact on quality of life and psychosocial and sexual well‐being.^3^, ^4^ In addition, a small proportion (3%–5%) develop malignancy.^5^, ^6^, ^7^

First‐line treatment with super‐potent topical corticosteroids is the accepted evidence‐based therapy.^8^ It is unusual for female patients not to respond to topical steroids, and if there is ongoing active disease, it is important to investigate for another cause. Lack of compliance, secondary infection, contact dermatitis to treatment, development of vulval neuropathic pain and most importantly, progression to differentiated vulval intra‐epithelial neoplasia all need to be excluded. In males who have severe phimosis or meatal involvement, surgical treatment would be required as topical steroids would not help this. The most effective second‐line therapy is unclear.

*‘What surgical treatments should be offered for lichen sclerosus?’* ^9^ was a research question prioritized during the Lichen Sclerosus Priority Setting Partnership in 2018. Surgical treatments were defined to include (but not limited to) laser, platelet‐rich plasma and lipofilling.

Laser is an emerging treatment for genital LS. Despite uncertain efficacy, its use is gaining popularity. Statements have been issued by international bodies to warn that lasers used for vaginal rejuvenation can cause serious harm^10^ and laser should not be used to treat LS outside the context of well‐conducted clinical trials.^11^

The aim of this systematic review (PROSPERO database registration‐CRD42019143039)^12^ was to establish the effectiveness of laser therapy for genital LS affecting men, women and children. All types of laser (any regimen, any time frame) were considered; the comparator was either active or placebo and the main outcomes assessed were (i) clinical signs, (ii) patient‐reported symptoms, (iii) quality of life and (iv) sexual function.

### 2.1 Patients and methods

Ovid MEDLINE, PubMed, Ovid Embase, Cochrane CENTRAL, Web of Science, CINAHL and PsycINFO were searched from inception to February 2021. PubMed search strategy used the following terms: (‘LS’ or ‘lichen sclerosis’ or ‘lichen sclerosus et atrophicus’ [MeSH Terms] or ‘vulvar lichen sclerosus’ [MeSH Terms] or ‘balanitis xerotica obliterans’ [MeSH Terms] or ‘balanitis xerotica’ or BXO) AND (laser or lasers or ‘lasers’ [MeSH Terms] or ‘laser therapy’ [MeSH Terms])

Randomized controlled trials (RCTs), non‐randomized designs, cohort studies or case series with five or more participants were included. Studies including photodynamic therapy using laser as the source of light and articles in languages other than English were excluded.

An information specialist conducted the searches, screening took place independently by three authors, and data extraction/quality assessment were performed independently by two reviewers with a third reviewer to resolve any disagreements. Quality assessment was performed using published tools where available for the identified study types: the revised Cochrane risk‐of‐bias tool^13^ (RCTs), ROBINS‐I tool^14^ (non‐randomized trials) and The Joanna Briggs Institute checklist^15^ (case studies). Data were extracted into an Excel spreadsheet (Table 1 summarizes the data and full details of what was extracted can be found on PROSPERO).^12^ Reference lists of the included studies were also reviewed as well as the clinicaltrials.gov website.

**TABLE 1:** Summary of characteristics and findings of the included studies

Study design	Study (Stated if Abstract Only)	*n*	Sex	Intervention (I) (vs. Comparison (C))	Results for Outcomes (i) Clinical Signs, (ii) Patient Reported Symptoms, (iii) Quality of Life (QoL) and (iv) Sexual Function	Conflicts of Interests (CoI)/Funding	Duration of Study and Follow up	Overall Risk‐Of‐Bias Judgment/Limitations Outlined
RCT	Belotto et al., 2016 *Abstract*	20	F	I‐ Diode laser once a week for 4 weeks. C‐ topical clobetasol propionate od for 4 weeks	(i) Increase in thickness of skin by 49.07% (*p* = 0.006) versus reduction in thickness in the control group (−27.51%) (ii) itch ‘decreased 50%’ versus. 83.76% in TCS group	FAPESP grant n82015/05259‐8	4 weeks of treatment, follow up from Dec 2014 to Sept 2015 – 10 months	High risk of bias using Cochrane risk of bias tool. Does not mention how LS was measured and confirmed.
RCT	Belotto et al., 2019 *Abstract*	12	F	I‐ Diode laser once weekly for 8 weeks. C‐ topical clobetasol propionate OD for 4 weeks, then alternate days for 4 weeks	(ii) Reduced itch in both groups (no numbers given). *PBMG inflammatory infiltrate decrease and collagen matrix remodeling were observed post‐treatment biopsy compared to pre‐treatment biopsy*.	Not stated by authors	8 weeks of treatment, no follow up stated	High risk of bias using Cochrane risk of bias tool
RCT	Bizjak Ogrinc et al., 2019	40	F	I‐ Non‐ablative neodymium: yttrium aluminum garnet (Nd:YAG) laser, 3 treatments every 14 days. C‐ Betamethasone diproprionate for 4 weeks decreasing regime: bd during first 2 weeks, od during the third week, and alternate days during the fourth week	(i) No significant difference in improvement of clinical photographs from baseline and 3 months after treatment (0.58 +/− 1.25 in the laser group and −0.27 +/− 1.27 in the control group, *p* = 0.8). (ii) Reduced itching, burning and pain in both groups compared with baseline rated on a visual analogue scale. Statistically significantly better in the laser group at 3 months, and authors state the effect was still significant at 6 months, although these symptoms started to increase again. (iv) The effect of LS on the quality of the patient’s sex life was reduced significantly only in the laser group. The problems recurred at 6‐month follow‐up.	Authors declared no CoIs	Duration of study 6 weeks, follow up 1 month after the start of therapy, then 3 and 6 months after the last treatment	High risk of bias using Cochrane risk of bias tool
RCT	Mitchell et al., 2020	40	F	I‐ Fractional CO_2_ laser‐ 5 treatments 4 weeks apart. C‐ sham treatment‐ 5 sessions	(i) There was a 6.82‐point reduction (improvement) in the patients’ Clinical scoring system for vulvar lichen sclerosus (CSS) from baseline in the active (95% CI = −11.28, −2.37, *p* = 0.004) and 4.83‐point reduction in the sham treatment group (95% CI = −9.16, −0.51, *p* = 0.03). In the clinicians’ CSS, there was a 0.82 increase (worsening) in the active (95% CI = −0.46, 2.11, *p* = 0.20) and a 0.28 reduction in the sham treatment group (95% CI = −1.53, 0.97, *p* = 0.65). *Primary outcome was histology. There was a* 0.12 *reduction (improvement) in Histopathologic Scale (HS) from baseline in the active treatment group (*95%CI = −1.01, 0.78, *p* = 0.79*) and a 0.06 increase from baseline in the sham treatment group (*95%CI *– −*0.81, 0.92*, p* = 0.90*). The change in HS between the active and sham arm was not statistically significant* (−0.17*;* 95%CI = *−*1.14, 1.06*, p* = 0.78).	Partial funding declared by authors. Funding by the manufacturer of the laser used in this study (Eluent group) but they had no role in the analysis and interpretation of data; in the writing of the report; or in the decision to submit the article for publication.	24 weeks treatment period and follow up 8 weeks post treatment	High risk of bias using Cochrane risk of bias tool
RCT cross over design	Siddique et al., 2020 *Abstract (Note previously published as a meeting abstract as Burkett et al, 2020)*	55	F	I‐ Fractional CO_2_ laser – no details on regime. C‐ Topical clobetasol propionate‐ regime not stated. Then crossed over at 6 months if still symptomatic	(iii) The greatest difference in Skindex‐29 overall score was in the laser group (−23.2, 19.0SD), especially in the baseline to 6 months. At 12 months the lowest scores were in the laser only group and highest in the steroid group. The steroid to laser group had scores similar at 6 months of steroid as did the steroid only group at 12 months. The steroid to laser group had a greater decrease in overall score with laser −1.52 (10.62 SD) versus −1.52 (10.62 SD) with steroid. There was a greater increase in patients who were 'satisfied or very satisfied on global impression of satisfaction (PGI‐S) in the steroid to laser group (52%) versus the laser to steroid group (22%) *p* = 0.001	R.E. Gutman: Boston Scientific; consultant and grant/research support, UpToDate; Royalty, Johnson & Johnson; Expert Witness Sling Class Action. A.Park: Allergan‐ speaker’s bureau. C.Iglesia: Foundation for female health awareness made payable to MedStar health Research Institute, grant/research support	6 months follow up after treatment, then 21/48 crossed over and were followed up for 6 more months (12 month data for 48 females).	High risk of bias using Cochrane risk of bias tool
RCT	Zhang et al., 2020	12	F	I‐ Fractional ultra‐pulsed CO_2_ laser‐ lesions were scanned by the laser 3–4 times in one treatment until the skin turned slightly red. C‐ high‐intensity focused ultrasound	(i) Colour and elasticity and ii) pruritis – ‘cured’ in 1 in laser group and 0 in ultrasound (US) group; ‘basically disappeared’ in 3 in laser group and 2 in US group; ‘improved’ in 1 in the laser group and 2 in US group; and ‘ineffective’ in 1 in the laser group versus 2 in US group.	Funding: Jiangsu Provincial Youth Medical Key Talent Project (QNRC2016303); Key project of Science and Technology Development Fund of Nanjing Medical University (2017NJMUZD045); General scientific research project of Jiangsu Provincial health Commission (H2018018)	Not stated	High risk of bias using Cochrane risk of bias tool
Non‐randomized trial	Li 2018 *Abstract*	63	F	I‐ Fractional CO_2_ laser‐ 3 times. C‐ Triamcinolone acetonide cream for 6 weeks	(i) – Skin elasticity score statistically lower than TCS at sixth month after treatment (*p* < 0.05). Recovery rate of submucosal haemorrhage (93.10%) was higher than TCS group (74.19%, *p* < 0.05). (ii) pruritus, (iv) dyspareunia score was statistically different after and before treatment in both groups (*p* < 0.05).	Not stated by authors	Duration of treatment not stated. Follow up 6–19 months	Serious risk of bias using the ROBINS‐I tool
Single arm trial	Baggish et al., 2016	27	F	I‐ Fractional CO_2_ laser – 3–4 treatments at 4–6 week intervals	(i) Visible improvement of skin colour, elasticity, vascularity was seen in 26/27 patients at one month following the 3rd treatment. (ii) No further itching or vulvar discomfort or pain in 24/27 women	States no financial conflicts exist	Duration up to 6 months and follow up at 12 months	Not blinded therefore observer bias. Application of topical nystatin‐triamcinolone for 48 h post Rx.
Single arm trial	Coyle et al., 2018 *Abstract*	7	F	I‐ Ablative fractional 2940nm Er:YAG laser‐ 3 treatments scheduled 4 weeks apart	(i) Photographs taken at baseline and at follow up (no further info). (ii) sig improved dryness, itching, burning, bleeding, soreness, easy tears, ulcerated lesions, (iii) QoL‐ 60% improvement at 1 month, 72% at 3 months (*p* = 0.007), (iv) sexual impact – 74% improvement at 1 month and 71% at 3 months (*p* = 0.07). *‘Three month biopsy data showed* 50%–100% *improvement over baseline in VLS symptoms’*.	Not stated by authors	Duration 3 months but no follow up after treatment	Reports findings of only 7 subjects out of 15. Follow‐up only to 3 months even though methods mention 6 months.
Single arm trial	Elias et al., 2018 *Abstract*	18	F	I‐ Patients with pre‐ dominant hyperkeratosis ‐ pixel ablative laser completing the treatment with autologous plasma in form of i‐PRF (injectable platelets rich in fibrin) and coverage of the lesion with l‐PRF (fibrin membranes rich in platelets); In predominant atrophic – subablative pixel CO_2_ laser and completed with i‐PRF dermic infiltrate. Three therapies, one every 30 days. All patients received immunity modulation with nutrition factors	(ii) and (iv) vaginal health Index (VHI) and Female sexual Function Index score (FSIS) questionnaire – 9 patients (50%) were asymptomatic. 4 patients manifested reduced itching and dyspareunia. One patient continued with severe dyspareunia but her itching got better. Nine patients had sexual intercourse of whom 75% (7 patients) had a positive response.	Not stated by authors	Duration 3 months but no detail on follow up	Only study included that used laser with additional treatment‐ autologous plasma and immunity modulation and therefore results not comparable with the other studies.
Single arm trial	Ferrara et al., 2020	10	M	I‐ Fractional CO_2_ laser‐ 3 sessions	(Fractional CO_2_ laser treatment improved the scores of all scales from baseline to day 280+/−10, significant at various time points for: (i) MenLas Observer scale – degree of reduced elasticity, atrophy and fissures (ii) symptoms of LS (MenLas Patient scale), (iii) DLQI test and (iv) sexual function (MSHQ questionnaire).	The authors report no conflict of interest.	Duration 100 days +/− 10. Follow up for 6 months after the last treatment.	An antibiotic cream was applied at the end of each session, and it was prescribed to be continued for five days after discharge. Small sample size. No randomization or control.
Single arm trial	Gomez‐Friero et al., 2019	28	F	I‐ non‐ablative, thermal‐only Er:YAG laser ×3 sessions every 4 weeks	(i) Sig improvement of ecchymosis, excoriations, and hypopigmentation. Improvement in labial fusion and hyperkeratosis (not stat sig). No improvement was seen in effacement. ii) sig reductions in itching, pain, (iii) improvement of impact of LS on patients’ lives (before treatment median score 10/11 and after treatment median score 4/11, *p* < 0.001)	No COI or funding	Duration 3 months. Follow up one month after the last session.	No skin biopsy to confirm LS diagnosis. No blinding of attending physician assessing clinical signs.
Single arm trial	Javaid et al., 2019 *Abstract*	11	F	I‐ Fractional CO_2_ laser ‐ up to 5 monthly treatments (mean 4)	(i) Improved whitening 82% (9/11), parchment‐like skin 73% (8/11), elasticity 70% (7/10) and lichenification 63% (5/8). Moderate to severe labial fusion improved in only four of the 11 subjects (36%). Ulcerations completely resolved in 3. (iii) improved quality of life. (iv) improved sexual func incl. dyspareunia (78%,6/8). Skin tearing worsened in 50% (3/6).	Candela Corporation provided partial funding for the study	Duration of study maximum 5 months, 3‐, 6‐ and 12‐month follow‐ups after the final treatment.	Not all had skin biopsy to confirm LS. Subjects maintained their existing topical steroid and exogenous hormone treatment, if any, during the study.
Single arm trial	Li et al., 2018 *Abstract*	42	F	I‐ Fractional CO_2_ laser ‐ total of 3 – 5 times, each time per‐month	(ii) vulvar pruritus, skin chapping, dyspareunia improved in 90.47% (38/42) (*P* < 0.001). The pruritus score of vulva (6.75 ± 1.79 vs. 1.78 ± 1.36) sig lower than that before treatment (*P* < 0.05), iv) Dyspareunia (4.88 ± 2.79 vs.2.15 ± 1.29, *P* < 0.05) improved sig. at one month. During 3 – 12 months follow‐up, 6/13 cases recovered sex.	Not stated by authors	Duration max of 5 months. Follow up 3 – 12 months	No detail on how LS was confirmed? biopsy. No detail on LASER dose. Limited detail.
Single arm trial	Pagano et al., 2020	40	F	I‐ Fractional microablative CO_2_ laser‐ 2 cycles at an interval of 30 or 40 days.	Significant improvement in: (ii) vulvar itching (iv) superficial dyspareunia and sensitivity during intercourse after two CO_2_ laser cycles.	Authors declare personal fees for lectures from Merck outside the submitted manuscript.	Duration max 80 days. Follow up 3 months after each treatment (median follow‐up of 5 months).	Short follow up
Single arm trial	Windahl et al., 2006	62	M	I‐ CO2 laser – no details on regimen given	(i) Forty men (80%) had no visible lesions and (ii) no local symptoms at follow up (range 5 months to 19 years). Ten patients had minor residual symptoms but only two required further treatment.	Not stated by authors	No detail of duration. Median follow up 14 years.	Not all patients were alive at follow up. Contact by email or telephone and only symptomatic patients invited to clinic for clinical examination. No detail on outcomes i.e., what local symptoms did patients have to begin with. No stats.
Case series ≥5	Balchander and Nyirjesy, 2020	40	F	I‐ Fractional CO_2_ laser‐ 2 or more laser sessions, spread at least 4 weeks apart	(i) White epithelium decreased by 20%. (ii) 72.5% described improvement as significant or more than 66% improvement. Statically sig. reduction in vaginal pain, itching, dysuria and (iv) dyspareunia	One author serves as a consultant for Hologic Inc, which owns the US license for the MonaLisa Touch laser	2 month duration. At 6 months after final treatment, follow‐up data are available for 29 patients (72.5%). Data are available for 22 patients (55%) at the 12‐month follow‐up.	Only 17 patients (42.5%) were confirmed to have LS through biopsy. Confounding factors: 35/40 (87.5%) used vaginal oestrogen too; patients still used TCS during laser treatment therefore difficult to know if effect was from TCS or laser therapy. One examiner (observer bias). Lost patients to follow up ‐ data are available for 22 patients (55%) at 12‐month follow‐up.
Case series ≥5	Dell et al., 2016 *Abstract*	15	F	I‐ Fractional CO_2_ laser ‐ 3 treatment sessions at 6 week intervals	(ii) Mean improvement of symptoms was 65% with a range of 10% to 100% (15/15) – 14/15 patients reported they were very satisfied or satisfied.	Not stated by authors	Duration 18 weeks. Follow up at 6 weeks after all 3 treatment sessions	Lack of detail in abstract. Follow up at 6 weeks only too short to conclude definite successful outcome.
Case series ≥5	Gardner et al., 2020	31	F	I‐ Fractional CO_2_ laser‐ 3 treatments, 6 weeks apart	(ii) VSQ vulvovaginal symptom questionnaire showed 18 out of 21 questions significantly improved (*P* < 0.05). (iv) all FSFI Female sexual Function Index score improved (pre 12.7 and post 19, *p* < 0.001); VAS, visual analogue scale showed significant improvement in painful intercourse (pre 6.6, post 2.4, *p* < 0.001) and vulvar dryness (pre 4.6, post 1.5, *p* < 0.001). Post treatments 17 additional women became sexually active.	No COI or funding declared	Duration 18 weeks. Mean follow 13.8 weeks.	Includes vulval atrophy and LS and difficult to identify data for LS alone and concomitant topical oestrogen was reported in 53%
Case series ≥5	Kartamaa et al., 1997	7	2 F 5 M	I‐ CO_2_ laser – no detail on frequency	(i) All penile lesions were clinically cured by laser treatment: however, urethral lesions of one patient recurred despite three separate treatments. The female patients with perineal LS improved significantly after the treatment, LS recurred in one patient.	Not stated by authors	Duration‐ no detail. Mean follow‐range 3 months‐ 6 years.	Follow up varied from months to years (3–6 years). Lack of detail‐ 'cured' but what are the specific outcomes?
Case series ≥5	Di Meo et al., 2018	8	3 F 5 M	I‐ Blue diode laser‐ total of 9 laser sessions, 3 sessions per week for 3 consecutive weeks.	(i) Clinical signs decreased from 7.75 to 3.5 with highest improvements in erythema, oedema and erosions. (ii) symptoms score decreased from 5.25 to 2.75. (iii) DLQI average score decreased from 5.75 to 3.75. (3‐point Likert scale used)	Authors declared none	Duration 3 weeks. Follow‐up 30 days after the last session.	Follow up only 30 days after the last laser session. Does not mention how LS was measured and confirmed.
Case series ≥5	Lee et al., 2016	5	F	I‐ 4 had fractional CO_2_ laser and 1 had ablative CO_2_ laser for severe, hyperkeratotic VLS– no detail on frequency	All patients responded positively.	No COI declared. No comment on funding.	No detail on duration. Follow up ranged between 6 and 48 months.	No clear outcomes. Not all patients were symptomatic‐ 'Four patients were symptomatic prior to treatment with itch and dyspareunia and one patient was asymptomatic'. In between the CO2 laser treatments, the patients were managed with 0.05% clobetasol and relapse was prevented with topical clobetasol 0.05% ointment.
Case series ≥5	Stuart et al., 1991	7	F	I‐ Non fractional ablative CO_2_ laser, under general anesthesia as an inpatient	(i) 5 patients were free of visible recurrence of disease and (ii) 5 patients were free of itch	Not stated by authors	No detail of duration. Follow up ranged from 12 to 37 months.	Did not mention how the outcome of itch or clinical assessment was measured. Patients used topical testosterone previously and had not tried current recommended topical steroid regime.
Case series ≥5	Teodoro et al., 2019	10	F	I‐ Fractional CO_2_ laser –1–3 treatments at one‐month intervals.	(i) improvement in the appearance of the introitus and in elastic opening and closing. (ii) Pruritus disappeared in the 50% and reduced remarkable in the other 50%, with a media VAS from 8.5 ± 2.22 to 1.2 ± 1.47 (*p* < 0.001). Complete resolution of burning in 60% ‐ reduction in the VAS score (5.7 ± 2.21 to 0.8 ± 1.32). (*p* < 0.001). Little improvement in the urinary problems. (iv) 2/9 with dyspareunia had complete resolution. The other seven had a great improvement with a media VAS 6 ± 3.13 at the beginning to 2.3 ± 2.41 (*p* = 0.0025). *Post‐treatment histology revealed trophic epithelium with mild acanthosis and small areas with superficial hyperkeratosis*.	Not stated by authors	Duration max. 3 months. Follow up after 3 months post treatment.	Half had been treated with TCS previously therefore half had not. Would be better to have patients with similar baseline characteristics.

## 3 RESULTS

**FIGURE 1:**
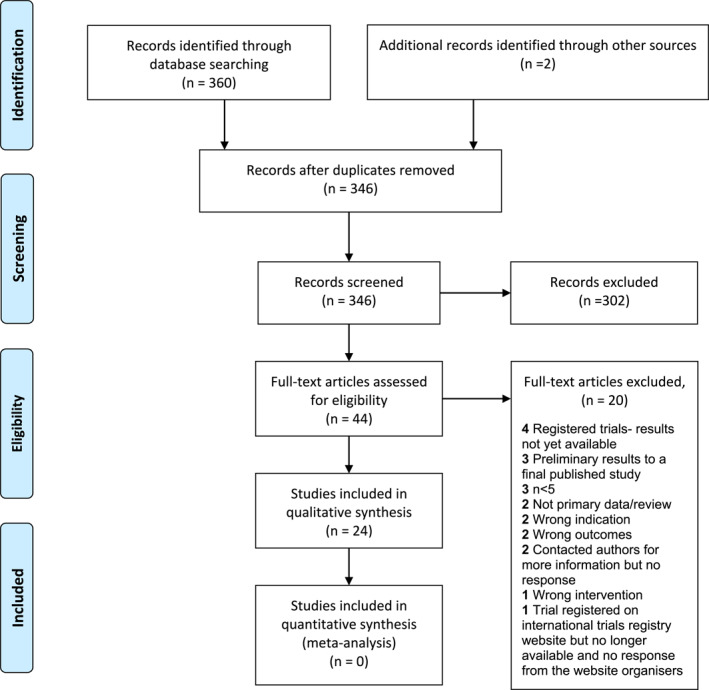
PRISMA 2009 Flow Diagram

Of 360 studies, 24 studies met the inclusion criteria, with a total of 610 participants (Figure 1) (women and/or men, but no children). The results of the studies are summarized in Table 1.

### 3.1 Randomized controlled trials

Of six identified RCTs,^16^, ^17^, ^18^, ^19^, ^20^, ^21^ three were conference abstracts^16^, ^17^, ^20^ and all included female participants. Laser was compared with either topical corticosteroid^16^, ^17^, ^18^, ^20^ sham laser^19^ or ultrasound.^21^

Four studies used different types of laser (diode laser, non‐ablative neodymium: yttrium aluminium garnet (Nd:YAG) laser and fractional CO_2_ laser) with differing regimens in women with biopsy proven LS (except one study which did not state how LS was confirmed),^16^ were compared against short courses (4–8 weeks) of superpotent topical corticosteroids.^16^, ^17^, ^18^, ^20^ A variety of outcomes were used in these RCTs; clinical signs such as skin thickness and histology, patient reported symptoms, quality of life scores and sexual function. Results suggest that patients allocated to receive laser had significant improvement in skin thickness (increased by 49.07% vs. −27.51% in the corticosteroid group, *p* = 0.006).^16^ There was no significant difference in improvement of clinical photographs from baseline and 3 months after treatment (0.58 +/− 1.25 in the laser group and −0.27 +/− 1.27 in the control group).^18^ Symptoms including itch, burning and pain rated on a visual analogue scale were reduced in both groups compared with baseline but they were statistically significantly better in the laser group at one and 3 month follow up and the effect was still significant at 6 months, although these symptoms started to increase again.^18^ Belotto et al., 2019 states decreased itch in both groups but no numbers are given^17^ and Belotto et al., 2016 states itch decreased less in the laser group compared to the topical corticosteroid group (by 50% vs. 83.76%).^16^ Improved Skindex‐29 score, a measure of quality of life, was demonstrated in both groups. At 6 months, the laser only group had the best reduction in Skindex score (baseline was 45.52 (15.2SD) and 23.1 (18.9SD) at 6 months) and the steroid only group had the least (baseline score was 51.22 (15.2SD), at 6 months it was 44.40 (17.0SD)). However, the score was similar at 12 months for the laser group (22.32 (23.4SD) versus 37.92 (22.7SD) for the topical steroid group). After crossing over at 6 months, the steroid to laser group had a greater decrease in score than the laser to steroid group and in terms of satisfaction measured by PGI‐S, the steroid to laser group were significantly more satisfied than the laser to steroid group, however there was no statistical comparison between the groups at any point.^20^ The impact of LS on the quality of patients’ sex life was reduced significantly only in the laser group but the problems recurred at 6‐month follow‐up.^18^

Mitchell et al. was the only RCT to compare CO_2_ laser with sham laser treatment. Forty women with LS were randomized in a 1:1 ratio to receive either five fractional CO_2_ treatments or five sham treatments in a 24‐week period. Histology was the primary outcome and although there was improvement in Histopathologic Scale (HS) in the active treatment group, the change in HS between the active and sham arm was not statistically significant (−0.17; 95% CI = −1.14, 1.06, *p* = 0.78). There was an improvement in the patient‐reported Clinical Scoring System for Vulvar Lichen Sclerosus (6.82‐point reduction from baseline in the active (95% CI = −11.28, −2.37, *p* = 0.004) and a 4.83‐point reduction in the sham treatment group (95% CI = −9.16, −0.51, *p* = 0.03). However, when the clinicians completed this scoring system, a 0.82 increase (worsening) was found in the active treatment group (95% CI = −0.46, 2.11, *p* = 0.20) and a 0.28 reduction in the sham treatment group (95% CI = −1.53, 0.97, *p* = 0.65), although the results were not significant. The authors conclude that CO_2_ laser is not an effective monotherapy treatment for vulvar LS but suggest it may be a useful adjunct to topical steroids.^19^

One RCT included 60 females with ‘white lesions of the vulva’ (12/60 had biopsy‐proven LS). Participants were randomized to either fractional ultra‐pulsed CO_2_ laser or high‐intensity focused ultrasound (US). Compared to focused US, clinical signs of colour, elasticity and symptoms of pruritis were ‘cured’ in 1 participant in the laser group and 0 in the US group; ‘basically disappeared’ in three in the laser group and two in the US group; ‘improved’ in one in the laser group and two in the US group; and was ‘ineffective’ in one in the laser group and two in the US group. Statistically improved outcomes across all patients with ‘white lesions of the vulva’ were stated for total cured (*p* = 0.01) and total effective rate (*p* = 0.02). The results for LS patients were not reported separately. Overall risk of bias using the Cochrane risk‐of‐bias tool was high for all of these RCTs.^21^

### 3.2 Non‐randomized trials

There was one non‐randomized trial comparing fractional CO_2_ versus topical triamcinolone acetonide for six weeks to treat vulval LS.^22^ The authors state the skin elasticity score in the laser group was statistically lower than that of the topical steroid group at 6 months after treatment (*p* < 0.05). Itch and dyspareunia were reported as being statistically significantly better after treatment in both groups but no further details were given in this abstract. Overall risk of bias using ROBINS‐I tool was high.

### 3.3 Single arm trials

Of nine single arm trials,^23^, ^24^, ^25^, ^26^, ^27^, ^28^, ^29^, ^30^, ^31^ seven were studies of women with vulval LS and two included men with genital LS.^26^, ^31^ The intervention was ablative Er:YAG laser in 1 study, non‐ablative Er:YAG in 1 study,^24^, ^27^ and fractional CO_2_ laser 6, with ablative CO_2_ in one. One study using fractional (sub‐ablative) CO_2_ had additional treatment with autologous plasma and immunity modulation with nutrition factors.^25^ The latter study states 50% of participants (9/18) were asymptomatic and 4 had reduced itching and dyspareunia although one continued with severe dyspareunia but her itching improved.

There were some clinical improvements observed including: visible improvement in skin colour, elasticity and vascularity, reduced elasticity, atrophy and fissures,^26^ significant improvement of ecchymosis, excoriations and hypopigmentation^27^ and improved whitening, elasticity and lichenification, although moderate to severe labial fusion improved in only four of the 11 subjects.^28^ Symptoms that were reported to improve include itching,^23^, ^24^, ^25^, ^27^, ^29^, ^30^ vulvar discomfort, pain or soreness,^23^, ^24^, ^27^ dryness, burning, bleeding, easy tears and ulcerated lesions.^24^ Quality of life was reported to improve in two studies^27^, ^28^ and some patients stated improved sexual function^25^, ^26^, ^28^, ^29^, ^30^ although skin tearing worsened in 50% (3/6) in one study.^28^

### 3.4 Case series

Of eight case series,^32^, ^33^, ^34^, ^35^, ^36^, ^37^, ^38^, ^39^ six included women with vulval LS and two included both men and women with genital LS.^13^, ^39^ All studies used CO_2_ laser except one which used blue diode laser.^36^ Improved clinical signs include decreased white epithelium by 20%,^32^ improved erythema, oedema and erosions^36^ no visible recurrence of disease (5/7)^38^ and improvement in the appearance of the introitus,^39^ however one study mentioned urethral lesions of one patient recurred despite three separate treatments (*n* = 5 males) and LS recurred in one female patient with perineum LS (*n* = 2).^35^ Studies report 65% improvement of symptoms (range 10%–100%),^33^ 18/21 questions assessing symptoms significantly improved,^34^ symptom score decreased from 5.25 to 2.75,^36^ there was a statistically significant reduction in vaginal pain, itch and dysuria,^32^ resolution of itch in 5/7 patients,^38^ pruritus disappeared in 50% and reduced in the other 50% as well as resolution of burning in 60%, however there was little improvement in urinary problems.^39^ One study reports DLQI score decreased from 5.75 to 3.75.^36^ Other studies report a statistically significant reduction in dyspareunia^32^ and 2/9 with dyspareunia had complete resolution.^39^

Meta‐analysis was not possible due to high heterogeneity of studies and low methodological quality.

## 4 DISCUSSION

We note a recently published systematic review^40^ which performed meta‐analysis on two of the RCTs which were also identified in our study (Bizjak 2019 and Burkett 2020).^8^, ^41^ The latter is a conference abstract, later published by Siddique et al in 2020.^20^ We assessed these studies as having high risk of bias. Outcome measures used to assess symptom score were very different (total symptom score vs. Skindex 29). The reports lacked the necessary detail required to independently calculate odds ratios and therefore, meta‐analysis was not possible. In addition, the systematic review by Li only looked at 13 studies; they did not include an additional 13 studies that we identified,^16^, ^17^, ^19^, ^21^, ^22^, ^25^, ^26^, ^28^, ^31^, ^33^, ^34^, ^35^, ^36^ four of which were RCTs.^16^, ^17^, ^19^, ^21^

There are many limitations of the studies identified in our systematic review. Conference abstracts (not peer reviewed) comprise 9 of the 24 included studies. Of the six RCTs, three were published in peer reviewed journals and only two^18^, ^19^ had a prospectively published protocol, despite all having been commenced after the introduction of mandatory trial registration guidelines.^42^ All RCTs identified were assessed to have a high risk of bias. In studies where the control group received topical steroids, the topical regimen was often shorter than 12 weeks, which is the recommended duration of treatment.^8^ The one non‐randomized trial (serious risk of bias) did not use super‐potent topical corticosteroids in the control group.^22^ The single arm trials are subject to considerable bias due to study type. Many studies are also under‐powered. Published case series lacked methodological detail and confounding factors, including concomitant use of topical treatments such as oestrogen during laser treatment were noted.^32^, ^37^ 7/24 of the studies were either commercially funded or declared conflicts of interest and 11/24 did not include conflicts of interest/funding statement.

Three studies included patients without biopsy‐proven LS and a further three did not state how LS diagnosis was confirmed, therefore there may be conditions which can mimic LS clinically included in these studies. The period of follow up after the last laser treatment was short in the majority of studies; between 1 month and 12 months in 16/24 studies; 11 of these followed up patients for 6 months or less. Three studies followed up the participants between 6 and 48 months^29^, ^37^, ^38^ and another study between 3 months and 6 years^35^ Three studies did not report the duration of follow‐up. Only one retrospective study had a long follow up period of 14 years; however not all patients were alive at follow up and those who were, were contacted by email or telephone and only symptomatic patients were invited to clinic for clinical examination to confirm the presence of no lesions.^31^

The limitations of this systematic review includes a lack of detailed data from some of the abstracts which published limited information and possibly the exclusion of negative studies due to publication bias. There are seven ongoing registered trials of which the results are awaited^43^, ^44^, ^45^, ^46^, ^47^, ^48^, ^49^; five of which are RCTs.^44^, ^46^, ^47^, ^48^, ^49^

In summary, there is no high‐quality evidence to support the use of laser for the treatment of genital LS in males and females. Long‐term data of laser are lacking including its adverse side effects. High quality RCTs are needed.

## CONFLICT OF INTEREST

None to declare.
